# Comparison of Ultrasound-Guided Vs Traditional Arterial Cannulation by Emergency Medicine Residents

**DOI:** 10.5811/westjem.2019.12.44583

**Published:** 2020-02-26

**Authors:** Casey Wilson, David Rose, Gabor D. Kelen, Veena Billioux, Leah Bright

**Affiliations:** Johns Hopkins Hospital, Department of Emergency Medicine, Baltimore, Maryland

## Abstract

**Introduction:**

We sought to determine whether ultrasound-guided arterial cannulation (USGAC) is more successful than traditional radial artery cannulation (AC) as performed by emergency medicine (EM) residents with standard ultrasound training.

**Methods:**

We identified 60 patients age 18 years or older at a tertiary care, urban academic emergency department who required radial AC for either continuous blood pressure monitoring or frequent blood draws. Patients were randomized to receive radial AC via either USGAC or traditional AC. If there were three unsuccessful attempts, patients were crossed over to the alternative technique. All EM residents underwent standardized, general ultrasound training.

**Results:**

The USGAC group required fewer attempts as compared to the traditional AC group (mean 1.3 and 2.0, respectively; p<0.001); 29 out of 30 (96%) successful radial arterial lines were placed using USGAC, whereas 14 out of 30 (47%) successful lines were placed using traditional AC (p<0.001). There was no significant difference in length of procedure or complication rate between the two groups. There was no difference in provider experience with respect to USGAC vs traditional AC.

**Conclusion:**

EM residents were more successful and had fewer cannulation attempts with USGAC when compared to traditional AC after standard, intern-level ultrasound training.

## INTRODUCTION

Arterial cannulation (AC) is often required in critically ill patients for continuous blood pressure monitoring, arterial blood gas sampling, and frequent blood draws.^1^,^2^ A common site for AC is the radial artery due to its superficial accessibility, safety due to the dual blood supply of the hand and relatively low complication rate.^1^–^3^ The palpation technique has long been the standard of care for inserting radial arterial catheters, but this technique can be difficult on obese, edematous, and hypotensive patients, leading to multiple attempts.^2^,^3^ Failure of this procedure can lead to hematomas, hemorrhage, and arterial vasospasm, which can compromise blood supply downstream.^2^, ^4^–^6^ These complications become more likely with increased number of cannulation attempts.^1^–^2^, ^7^, ^8^

Ultrasound (US) guidance to cannulate central and peripheral veins has proven successful, safe, and effective.^10^–^12^ It has become the standard of care for central line placement at most academic medical centers. Using US for arterial line placement has proven itself in the perioperative setting^4^, ^8^, ^13^–^16^ and in several systematic reviews.^9^, ^17^–^18^ In one systematic review, Shiloh et al demonstrated that “compared with the palpation method, ultrasound guidance for arterial catheterization was associated with a 71% improvement in the likelihood of first-attempt success.”

Shiver et al studied US-guided arterial cannulation (USGAC) in the emergency department (ED) and found USGAC was successful more frequently and took less time to establish the arterial line as compared with the palpation method.^2^ Two recent studies involving anesthesia residents found benefit using USGAC.^13^,^14^ However, these perioperative patient populations are typically more hemodynamically stable and AC is less urgent than in an emergency department (ED) setting. USGAC has never been studied with emergency medicine (EM) residents. Our study design mirrored that of Shiver et al, who performed their randomized controlled trial using US-credentialed attending “experts” as operators. We sought to reproduce their findings and validate their results but instead used residents, with standard US training, as US operators. This study sought to compare traditional AC to USGAC as performed by EM residents with varying skill levels in US. We hypothesize EM residents with standard US training will have more success with AC using US guidance vs traditional AC technique.

## METHODS

This study was a prospective, randomized, interventional study conducted at a tertiary care, academic urban ED with approximately 70,000 adult visits per year. Patients were enrolled over an 18-month (2014–2015) and an additional eight-month (2017) period to allow for additional enrollment in the study. ED patients over 18 years of age requiring AC for continuous blood pressure monitoring or frequent blood draws were enrolled in the study. Exclusion criteria were those with contraindications for radial arterial access, such as overlying cellulitis or bony injury, a pre-existing arterial catheter at an alternative site, or any other reason for exclusion for patient or staff safety at the discretion of the ED attending physician. Research was conducted in accordance with ethical standards of the institutional review board (IRB). The IRB determined that the study was a quality improvement effort because both forms of line placement were currently in use in the ED and in line with the standard of care; thus patient consent was waived.

Second- and third-year EM residents performed AC. Each participating resident completed a four-hour intern training session introducing interns to the focused assessment with sonography in trauma (FAST) exam, vascular access, cardiac, gallbladder, renal, musculoskeletal, thoracic and ocular US. Intern year includes a two-week intern US rotation. This rotation includes scanning sessions with US faculty, and weekly Q/A sessions and instruction. These sessions are standard to the curriculum at our residency; there were no deviations or changes when the study started. This training reflects that residents underwent basic US training as set forth by the 2016 American College of Emergency Physicians (ACEP) Ultrasound Guidelines.^19^ These guidelines stipulate the number of US needed in each US competency to qualify for graduation. They all completed at least five US-guided vascular access procedures, and the images were reviewed by the institution’s emergency ultrasound director. Residents are required to complete this requirement at the completion of their intern year.

Quality assurance of all procedures is reviewed by the US director, and all invasive lines are supervised by an attending physician. Eligible patients were randomized by the last digit of their medical record number (MRN). If the last digit of the patient’s MRN was odd, traditional AC by palpation was performed; if the last digit of the MRN was even, US-guidance was used. Standard Arrow (Teleflex, Morrisville, NC) 20-gauge arterial catheters were used for all procedures.^20^ Both Sonosite M-turbo US (Fujifilm Sonosite, Bothell, WA) with 13-6 megahertz (MHz) linear transducers and Philips Sparq (Koninklijke Philips, Amsterdam, the Netherlands) US with 12-4 MHz linear transducers were available for USGAC.

**Population Health Research Capsule**

What do we already know about this issue?

Ultrasound (US) is well established in the literature as a procedural adjunct that enhances patient safety and successful placement for central venous catheters.

What was the research question?

Can US novices duplicate this success with arterial lines?

What was the major finding of the study?

Emergency medicine residents with minimal training were able to successfully thread arterial catheters with fewer attempts when they used US.

How does this improve population health?

Physicians should consider using US when available to decrease the number of painful arterial sticks in critically ill patients.

Patients were prepped for an arterial line in the standard sterile fashion with patient supine, wrist extended and hand fixed with adhesive tape. For the USGAC group, the US machine was prepped with a sterile probe cover. The procedure duration was timed by an available attending, nurse, or ED technician; for both techniques, the stopwatch began when the needle punctured the skin. Once the skin was punctured, the resident could redirect the needle, but each time the needle exited and re-entered the skin it was considered an additional attempt. An attempt was successful and time was stopped when pulsatile blood was returned through an advanced arterial catheter, at which point the line was secured. To minimize the possibility of numerous failed attempts at AC, we limited access attempts in each group to three per patient. If they were not successful after three attempts the clock was stopped, time was noted, and they crossed over to the alternative technique as a rescue maneuver to achieve line placement and for patient comfort.

Patient demographic data, heart rate, blood pressure, and characteristics such as vasopressor use and intubation were noted. We recorded data on the number of attempts, time of the procedure in seconds, whether the catheter was successfully placed, or whether they needed to cross over to the alternate technique. Complications at the time of the placement were also noted; these included lacerations, arterial occlusion, and hematoma. Additionally, a record was kept of the degree of previous experience that each subject had with palpation and US-guided arterial lines. All data was stored on a protected institutional server.

The primary outcome variable was the number of attempts needed for successful arterial catheter placement. The secondary outcome variables included time zero to arterial catheter placement and number and type of complications.

Sample-size calculations were based on prior studies. We compared the palpation and US-guided groups using descriptive statistics. The number of attempts needed for successful arterial catheter placement, and time to successful placement was compared using t-tests after normality and variance were assessed. Comparison of the proportion successful and with complications was done using χ^2^ tests. We analyzed data using STATA 15.0 (StataCorp, College Station, TX).

## RESULTS

A total of 60 patients were enrolled into the study, with 30 randomized to the palpation group and 30 to the US-guided group. Demographic information and indications for AC can be found in Table 1. There were no significant differences between the two groups with respect to any demographic information, clinical characteristics, or arterial line indication (p<0.05). Sixteen (53%) patients in the palpation group required rescue with US guidance and one (3%) crossed over from the US group to palpation (Table 2). An arterial line required a mean of 1.3 attempts in the US group vs 2.0 attempts in the palpation group (p<0.001). An arterial line was successfully placed in 29 (96%) of the US group vs 14 (47%) in the palpation group (p<0.001). Of the 16 failed traditional AC that crossed over to USGAC, there was 100% (16/16) success rate with USGAC rescue. We found no significant differences in the time it took for placement or the complication rate between the two arms. There was no significant difference in the providers’ prior experience with respect to USGAC vs traditional AC (Table 3).

## DISCUSSION

The study hypothesis stated EM residents with standard US training would be more successful using US guidance for AC than using the traditional palpation technique. This study reproduced the findings and validated the results by Shiver et al, who used US-credentialed faculty instead of residents to illustrate that USGAC was more successful than the palpation technique for placing arterial lines.^2^ Our results indicate US is safe, has a high success rate, and can be performed proficiently after standard training. In our clinical experience, USGAC is often used as a back-up when traditional palpation techniques failed. This often occurs with critical patients who are hypotensive, obese or, edematous. The success rate of initial arterial line placement with US over palpation alone is significant (96% vs 47%). Also notable is the percentage of arterial line placements randomized to the palpation technique that converted to US rescue for successful placement (53%). One may argue that the difference between the groups may be because the residents are not good at placing arterial lines by palpation. However, as outlined in Table 3, there was no difference with respect to residents’ prior experience with placing arterial lines with or without US.

The ACEP 2016 Emergency Ultrasound Guidelines and several sources routinely highlight the safety and efficacy of US guidance for central venous access, but AC is not universally noted or included.^20^ Along with central line placement, US-guided arterial line catheterization should be taught and considered standard of care for physicians who have undergone standard US training for vascular access. Additionally, these findings have implications for other specialties with less standardized or formalized ultrasound education. If novices can do this quickly and successfully, one could conclude this would prove useful in the intensive care and perioperative settings as well.

## LIMITATIONS

This study was limited by its single-site enrollment. Due to the critically ill nature of the patients requiring AC, enrollment was likely lower overall and took longer to complete. Our enrollment period was extended to enroll more patients and improve statistical calculations. The gap between enrollment periods was due to new researchers adding to the project. There were no changes to methodology or resident US training during this time period. In a busy ED it often was not feasible to remember to enroll patients for randomization; oftentimes, the procedure needed to be performed emergently. We have a convenience sampling of patients, and sampling bias was involved. Additionally, the 24/7 nature of enrollment meant that the timekeepers were not formally trained and no inter-rater reliability testing could be validated. This may have influenced overall time calculations, likely on the extremes. Future, larger studies are needed to validate our results.

## CONCLUSION

This study demonstrated EM residents were more successful and had fewer cannulation attempts with ultrasound-guided radial arterial cannulation when compared to the traditional AC method after standard, intern-level US training. We conclude that using US guidance for AC requires standard training and can be useful for physicians and improve quality of care and safety for their patients.

## Figures and Tables

**Table 1:** Patient demographics, clinical characteristics and indications for arterial line placement.

	Arterial line placement		
	Overall (n=60)	US guided (n=30)	Palpation (n=30)
Gender, n (%)			
Male	32 (53.3)	15 (50.0)	17 (56.7)
Female	28 (46.7)	15 (50.0)	13 (43.3)
Age, mean (SD) (Missing = 1)	61.2 (±16.75)	62.4 (±16.09)	60.0 (±17.61)
BMI, mean (SD) (Missing= 8)	27.3 (±7.75)	27.3 (±8.25)	27.4 (±7.39)
HR, mean (SD) (Missing =1)	87.8 (±27.79)	89.7(±30.77)	85.8(±24.73)
MAP, mean (SD)	92.1(±40.18)	99.7 (±42.57)	84.8 (±36.99)
SBP, mean (SD)	127.9(±59.28)	133.6 (±62.39)	122.1 (±56.37)
Intubated, n (%)			
Yes	30 (50.0)	18 (60.0)	12 (40.0)
No	30 (50.0)	12 (40.0)	18 (60.0)
Pressors, n (%)			
Yes	15 (25.0)	7 (23.3)	8 (26.7)
No	45 (75.0)	23 (76.7)	22 (73.3)

	Indications for arterial line placement		

BP Monitoring, n (%)			
Yes	54 (90.0)	27 (90.0)	27 (90.0)
No	6 (10.0)	3 (10.0)	3 (10.0)
ABG Sampling, n (%)			
Yes	17 (28.3)	10 (33.3)	7 (23.3)
No	43 (71.8)	20 (66.7)	23 (76.7)
Frequent Blood Draws, n (%)			
Yes	7 (11.7)	4 (13.3)	3 (10.0)
No	53 (88.3)	26 (49.1)	27 (90.0)

*US*, ultrasound; *SD*, standard deviation; *BMI*, body mass index; *HR*, heart rate; *MAP*, mean arterial pressure; *SBP*, systolic blood pressure; *ABG*, arterial blood gas.

**Table 2:** Mean number of attempts at arterial line placement, number of successful attempts, mean time to complete the procedure successfully, and number of complications.

	Arterial line placement		
	US Guided (n = 30)	Palpation (n = 30)	P-value
Attempts, mean (±SD)	1.3 (±0.596)	2.0 (±0.928)	<0.001
Success, n (%)			<0.001
Yes	29 (96.7)	14 (46.7)	
No	1 (3.3)	16 (53.3)	
Time (seconds), mean (SD)	235.9 (±203.4)	249.1 (±255.0)	0.83
Complications, n (%)			0.15
Yes	6 (20.0)	11 (36.7)	
No	24 (80.0)	19 (63.3)	
Complication type			0.36
Hematoma	5 (16.7)	9 (30.0)	
Laceration	0 (0.0)	0 (0.0)	
Occlusion	1 (3.3)	2 (6.7)	
None	24 (80.0)	19 (63.3)	

*US*, ultrasound; *SD*, standard deviation.

**Table 3:** Provider experience: the number of arterial lines placed using ultrasound and palpation by residents in their career, and number of residents by postgraduate year in each group.

	Overall (n = 60)	US Guided (n = 30)	Palpation (n = 30)	P-value
US-Guided Experience; n (%)				0.07
<10 A lines	21 (35.0)	9 (30.0)	12 (40.0)	
10–30 A lines	30 (50.0)	19 (63.3)	11 (36.7)	
>30 A lines	9 (15.0)	2 (6.7)	7 (23.3)	
Palpation Experience, n (%)				0.38
<10 A lines	27 (45.0)	15 (50.0)	12 (40.0)	
10–30 A lines	27 (45.0)	11 (36.7)	16 (53.3)	
>30 A lines	6 (10.0)	4 (13.3)	2 (6.7)	
Resident Level, n (%)				0.07
PGY2	31 (51.7)	12 (40.0)	19 (63.3)	
PGY3	29 (48.3)	18 (60.0)	11 (36.7)	

*US*, ultrasound; *PGY*, postgraduate year; *A*, arterial.
